# Challenges and Opportunities of Anti-Bullying Intervention Programs

**DOI:** 10.3390/ijerph16101810

**Published:** 2019-05-22

**Authors:** Peter K. Smith, Sheri Bauman, Dennis Wong

**Affiliations:** 1 Department of Psychology, Goldsmiths, University of London, London SE14 6NW, UK; 2 Department of Disability and Psychoeducational Studies, University of Arizona, Tucson, AZ 85721, USA; sherib@email.arizona.edu; 3 Department of Social and Behavioural Sciences, City University of Hong Kong, Hong Kong, China; dennis.wong@cityu.edu.hk

Over recent decades, bullying, and the more recent version of cyberbullying, have come to be recognized as important social and public health issues, generating an increasing volume of publications. It has been understood as a global, international issue, as evidenced in the UNESCO 2017 report on School Violence and Bullying: School Status Report. It has mainly been studied in schools, but it can occur in many contexts. Wherever it occurs, it can have harmful and pernicious effects, either short-term or long-term, for all involved including bystanders. For the victims especially, outcomes can include loss of self-esteem, depression, suicidal tendency, health problems, and reduced academic or work performance. Perpetrators, if unchallenged, can learn that this kind of abusive behavior can be carried out with impunity and continue on a pathway of antisocial behavior.

Most scholarship on bullying references the social-ecological model, which situates the individual at the center of concentric circles representing the various contexts in which the individual develops: Family and school, neighborhood, community and society, and historical time. As we move forward in this field, we need to examine how those larger, more distant layers impact the work done in schools.

From the origins of research on bullying, there have been attempts to intervene, noticeably in school settings. These are having some success, as evidenced by meta-analyses [1] and by collected contributions from across the globe [2,3]. An important phase in current research is to document successes and failures in anti-bullying interventions, and relate these to our rapidly growing knowledge base.

This Special Issue contains 14 contributions on the topic of interventions against bullying, including cyberbullying, and similar abusive behaviors, such as dating violence. There are also some papers in this Special Issue that assess positive or protective factors, such as well-being, self-efficacy, and school climate. Eight of the 14 contributions directly assess the effects of an intervention, with pre-/post-test designs and experimental and control groups. The other contributions examine a range of relevant topics, such as teacher attitudes, and pupils’ confidence in intervening rather than being passive bystanders. Ten countries are represented among the authors, although both Spain and Italy make several contributions. Most of the articles are about secondary schools (pupils or teachers), but there are also contributions on early childhood, primary school, and university.

Many issues are highlighted in the contributions, but here we mention a few. One important aspect in interventions is whether bystanders (who often form a ‘silent majority’) feel empowered to act in a more prosocial way and defend a victim [4]. As shown in [5], this may depend in part on the pupil’s own self-confidence, but also on wider aspects, such as class cohesion, or school climate. Victims too may or may not feel able to cope with bullying in effective ways; coping strategies can be enhanced as indicated in several studies [6,7,8].

Many interventions are broad-brush approaches given to a whole class or whole school. These can be valuable, but as shown in [9], they may be differentially effective for different pupils. Some pupils may need more targeted interventions, and it will be important to identify who they are as well as what kind of intervention is needed.

The actual interventions used at class or school level vary across particular programs [6,7,8,9,10,11,12,13]. However, they generally include some awareness-raising components, the role of bystanders, suggestions about coping strategies, and, in some cases, peer support elements in which other pupils can offer advice. However, for interventions against school bullying, teachers clearly have a vital and leading role; usually, they are delivering the anti-bullying programs. Several contributions focus on the teacher’s role, including their attitude and confidence [14] and how their actions may be perceived by parents [15], or pupils [16]. One contribution [8] focusses on martial arts training, suggesting that traditional approaches focusing directly on bullying have had rather little success. In fact, direct approaches do have some success [1], but there is a balance to be struck between more general preventative approaches, and specific anti-bullying work—which is found already in some programs such as KiVa [7].

Unfortunately, the abuse of power is always going to be a temptation for some individuals who are in a powerful position—whether in schools or in the wider society. It is not going to be possible to ‘eliminate’ bullying. However, we can take actions to reduce its prevalence, to provide more effective help for victims, to assist bystanders to act positively, to encourage perpetrators to change their ways, and generally to improve safety and climate in schools and other settings [17,18]. This is likely to be especially important in schools, generally seen as the preparation for adult life. 

On a more somber note, one of the corresponding authors, Anna Constanza Baldry, sadly died after her contribution [13] was accepted. Correspondence about this article should be addressed to one of her co-authors.
